# Cetyl Alcohol Polyethoxylates Disrupt Metabolic Health in Developmentally Exposed Zebrafish

**DOI:** 10.3390/metabo13030359

**Published:** 2023-02-28

**Authors:** Matthew K. LeFauve, Roxanne Bérubé, Samantha Heldman, Yu-Ting Tiffany Chiang, Christopher D. Kassotis

**Affiliations:** Institute of Environmental Health Sciences, Department of Pharmacology, Wayne State University, Detroit, MI 48202, USA

**Keywords:** endocrine disrupting chemicals, adipogenesis, alcohol ethoxylates, ethoxylated surfactants, mixtures, obesogen

## Abstract

Alcohol polyethoxylates (AEOs), such as cetyl alcohol ethoxylates (CetAEOs), are high-production-volume surfactants used in laundry detergents, hard-surface cleaners, pesticide formulations, textile production, oils, paints, and other products. AEOs have been suggested as lower toxicity replacements for alkylphenol polyethoxylates (APEOs), such as the nonylphenol and octylphenol polyethoxylates. We previously demonstrated that nonylphenol polyethoxylates induced triglyceride accumulation in several in vitro adipogenesis models and promoted adiposity and increased body weights in developmentally exposed zebrafish. We also demonstrated that diverse APEOs and AEOs were able to increase triglyceride accumulation and/or pre-adipocyte proliferation in a murine pre-adipocyte model. As such, the goals of this study were to assess the potential of CetAEOs to promote adiposity and alter growth and/or development (toxicity, length, weight, behavior, energy expenditure) of developmentally exposed zebrafish (*Danio rerio*). We also sought to expand our understanding of ethoxylate chain-length dependent effects through interrogation of varying chain-length CetAEOs. We demonstrated consistent adipogenic effects in two separate human bone-marrow-derived mesenchymal stem cell models as well as murine pre-adipocytes. Immediately following chemical exposures in zebrafish, we reported disrupted neurodevelopment and aberrant behavior in light/dark activity testing, with medium chain-length CetAEO-exposed fish exhibiting hyperactivity across both light and dark phases. By day 30, we demonstrated that cetyl alcohol and CetAEOs disrupted adipose deposition in developmentally exposed zebrafish, despite no apparent impacts on standard length or gross body weight. This research suggests metabolic health concerns for these common environmental contaminants, suggesting further need to assess molecular mechanisms and better characterize environmental concentrations for human health risk assessments.

## 1. Introduction

Alcohol polyethoxylates (AEOs) are high-production-volume nonionic surfactants used in laundry detergents (39% of total use), hard surface cleaners (13%), dishwashing detergents (12%), personal care products (23% of total use), and in a variety of other industrial and/or household applications [1]. The high efficiency and low costs of polyethoxylated surfactants have supported global annual production of >13 million metric tons in 2008 [2] and >33 billion USD in global revenues in 2014 [3]. Alkylphenol polyethoxylates (APEOs), such as nonylphenol ethoxylates (NPEOs), are widely used nonionic surfactants with growing evidence for toxicity. This encouraged a 2010 US Environmental Protection Agency (EPA) report: the Nonylphenol and Nonylphenol Ethoxylates Action Plan, which was designed to address concerns over ecological and other effects associated with the use of NPEOs. As part of this process, EPA’s Design for the Environment Program prepared an “Alternatives for Nonylphenol Ethoxylates” report in 2012 to help industry choose lower-toxicity replacement chemicals [4]. Despite the high reported aquatic toxicity, AEOs have been presented as some of the recommended alternatives, given their presumed lower persistence and lower metabolite toxicity [4,5]. Alcohol ethoxylates are thus the most widely used alternatives for APEOs in Europe and likely elsewhere [6].

As such, diverse AEOs have been widely reported globally at a range of concentrations in influent, effluent, surface and groundwater, and even in treated drinking water. Alcohol ethoxylates were measured in surface water near US wastewater treatment plants at mean concentrations from 116–184 μg/L in influent, from 286 to 506 μg/L in effluent and from 105 to 315 μg/L in outfall [7]. AEOs were measured in sewage treatment plants and receiving waters in NE Spain, with C_12_ ethoxylates ranging from <LOD to 12.3 μg/L (90% detection frequency), C_14_ ethoxylates ranging from <LOD to 19.4 μg/L (84% detection frequency), and C_16_ ethoxylates detected only in influent samples [5]. Concentrations of AEOs were measured in effluent from activated sludge wastewater treatment plants in Europe and Canada, with an overall mean concentration of 5.7 μg/L [8]. Specifically, median concentrations of C_12_, C_13_, C_14_, C_15_, C_16_, and C_18_ ethoxylates were 0.8 μg/L (14% of total AEO burden), 1.1 μg/L (19%), 1.0 μg/L (18%), 0.9 μg/L (17%), 1.0 μg/L (17%), and 0.9 μg/L (16%), respectively [8]. C_12_ AEOs were also reported in Polish sewage effluent at concentrations that decreased with increasing ethoxylation: 0.6–2.0 μg/L for the base alcohol, 0.2–0.5 μg/L for one ethoxylate chain, and down to 50–150 ng/L for C_12_ alcohols with a six ethoxylate chain [9]. Similarly, river water samples were reported from 300 to 850 ng/L for the base alcohol and 50 to 500 ng/L for the one-to-six ethoxylate chain length C_12_ alcohols [9]. C_12_ AEOs (3–9 ethoxylate chains) were reported in the groundwater of farming areas in Denmark at concentrations from 60 to 190 ng/L and in soil interstitial water from 30 to 70 ng/L [6]. Colorado River and Colorado drinking water samples were analyzed for a series of AEOs, reporting C_12_, C_13_, C_14_, C_15_, C_16_, and C_18_ AEOs with ethoxylate chains ranging from 2 to 20 at low ng/L concentrations [10]. Overall, these studies suggest widespread contamination with high ng/L to μg/L concentrations of diverse alkyl chain length and ethoxylate chain length AEOs across water types and regions.

Studies have generally described AEOs as readily degraded in the environment, though some have described only partial degradation, potentially due to the toxicity of the AEOs to the bacteria responsible for their degradation [11]. This research also demonstrated that AEOs were the most toxic surfactants tested across a range of species [11]. Comprehensive assessment of diverse C_12–18_ and EO_0–18_ AEOs and degradation through wastewater treatment plants (WWTPs) across the United States reported 95%+ removal. However, the retention of diverse AEOs in effluent samples was approximately 3.64 μg/L, suggesting massive influent concentrations of diverse AEOs [12]. While limited research has evaluated the in vitro biotransformation of shorter alkyl chain length AEOs in fish, the reported bioconcentration factors have indicated the potential for long-term adverse effects in aquatic environments [13]. An even higher bioconcentration factor was identified in follow-up research for an AEO with a longer alkyl chain length (equivalent to the one studied here), suggesting potential for bioaccumulation of AEOs [14].

Unlike APEOs, limited research has been conducted to investigate the in vivo health impacts of AEOs as they are presumed to be lower toxicity replacements [15,16,17,18,19,20,21,22,23]. We previously examined the adipogenic activity of a range of AEOs and APEOs in the 3T3-L1 adipogenesis model [24]. We demonstrated that both AEOs (including cetyl, lauryl, and tridecyl alcohol) and APEOs promoted significant adipogenic activity, with NPEO and cetyl alcohol ethoxylates (CetAEOs) inducing the greatest magnitude of effects via both triglyceride accumulation and pre-adipocyte proliferation. The adipogenic effects appeared to be specific to the ethoxylate chain length, with the base hydrophobes inducing limited or no adipogenic activity themselves and medium (4–10 ethoxylate chain lengths) size inducing maximal adipogenic activity [24]. Just recently, we confirmed these findings in vivo for the NPEOs [25], reporting consistent obesogenic effects on growth and development, particularly for the medium-chain-length NPEOs. However, we have yet to confirm these findings for the AEOs, such as the CetAEOs.

Given the increasing use of zebrafish for metabolic health research [26,27,28,29,30,31,32,33,34,35], similarities to humans [36,37,38,39], and calls for reducing mammalian vertebrate animal use, there is a strong impetus for the further utilization of the zebrafish model to conduct metabolic health assessments. Specifically, zebrafish have emerged as a validated model for metabolic health research [26]. They develop quickly and have morphologically similar adipose to humans, storing neutral triglycerides in lipid droplets within white adipocytes, similar to mammals, and have similar gene expressions associated with adipocyte differentiation, lipolysis, and endocrine function [36,38,39,40,41]. Zebrafish are transparent and are thus readily amenable to fluorescent staining and full-body imaging to characterize and quantify their 34 anatomically, physiologically, and molecularly distinct adipose depots [37,38,39], with a comprehensive developmental timeline available to assess perturbations in adipose deposition [38,39]. Given these factors, a growing body of literature has utilized zebrafish to demonstrate altered growth, adipose development, and metabolic dysfunction following exposure to diverse environmental contaminants [26,27,28,29,30,31,32,33,34,35].

Moreover, there is an expanding body of work identifying diverse environmental contaminants as metabolism disruptors able to directly modulate metabolic health endpoints in vitro and/or in vivo [42,43,44,45,46]. With metabolic disorders, such as obesity, vastly increasing in incidence (obesity affecting >42% of US adults, >70% obese and overweight [47]), it is imperative to characterize how metabolism-disrupting environmental contaminants may be exacerbating this pandemic.

The goals of this study were to assess the potential of CetAEOs to disrupt metabolic health in vivo. We hypothesized that medium ethoxylate chain lengths (6–10 ethoxymers) would promote the greatest adipogenic/obesogenic effects, consistent with what we previously observed for the NPEOs [25]. While we previously demonstrated adipogenic activity for a single CetAEO [24], the influence of varying ethoxylate chain lengths on adipogenic and/or obesogenic effects has yet to be systematically assessed. We sought to expand our understanding through the evaluation of CetAEOs in two separate human mesenchymal stem cell (hMSC) models and also in the murine 3T3-L1 pre-adipocyte model. We further evaluated CetAEOs through comprehensive metabolic health evaluation in a developmental exposure zebrafish model to assess in vivo metabolic health disruption.

## 2. Materials and Methods

### 2.1. Chemicals

The chemicals used are described in detail in Table 1. Stock solutions were prepared in 100% DMSO (Sigma, St. Louis, MO, cat # D2650) using the molecular weight (control chemicals) or average molecular weight (ethoxylated surfactants). Since none of the ethoxylates included here have commercially available pure standards, we instead utilized commercial mixtures with average ethoxylate chain lengths (Table 1). All of the stock and working solution vials were stored at −20 °C between uses. All of the chemicals were tested in vitro and in vivo at concentrations ranging from 10 μM to 1 nM, though 10 μM concentrations were toxic in vivo. Therefore, 1 μM is the highest test concentration used in zebrafish. (TBT: 1 nM–1 pM and MEHP: 1 and 0.1 μM for in vivo testing; Table 1).

### 2.2. Cell Care

3T3-L1 cells (Zenbio cat# SP-L1-F, lot# 3T3062104; Research Triangle Park, NC; passage 8) were maintained in pre-adipocyte media (Dulbecco’s Modified Eagle Medium–High Glucose; DMEM-HG; Gibco # 11995, Thermo Fisher, Waltham, MA, with 10% bovine calf serum and 1% penicillin/streptomycin; Gibco # 15140) at a subconfluent state, as described previously [48,49,50,51,52], and utilized between passages 8 and 12. 3T3-L1 cells were seeded at ~30,000 cells per well into 96-well tissue culture plates, grown to confluency, and then allowed 48 h for growth arrest and clonal expansion before initiating differentiation (Figure S1A). Differentiation was induced by replacing media with test chemicals and/or controls (Table 1) using a DMSO vehicle (at 0.1%) in differentiation media (DMEM-HG with 10% fetal bovine serum, 1% penicillin/streptomycin, 1.0 μg/mL human insulin, and 0.5 mM 3-isobutyl-1-methylxanthine, IBMX). After 48 h of differentiation induction, the media were replaced with fresh dilutions of the test chemicals and/or control chemicals in adipocyte maintenance media (differentiation media without IBMX), and these media were refreshed every 2–3 days until assay, ten days after induction.

Zenbio (cat# HBMMSC-F, lot# HBMMSC071819A, female, Caucasian, age 35) and Lonza (cat# PT-2501, lot# 19TL155677, male, Black, age 31; Lonza, Basel, Switzerland) hMSCs were induced to differentiate according to manufacturer’s instructions, as described previously [25]; differences in differentiation timelines reflect the differences in recommended protocols by the cell line providers (Lonza and Zenbio). Briefly, the cells were seeded in provider-specific basal media into 96-well plates at 10–15,000 cells per well and grown to confluence. Once confluent, differentiation was induced using the cell line providers’ commercially available differentiation media (Figure S1B,C). Briefly, the media were replaced with the test chemicals in differentiation media, as above (Zenbio catalog # DM-2-500; Lonza catalog # PT-3102B). For Zenbio-sourced cells, the differentiation media were left undisturbed for three days and then removed and replaced with fresh dilutions in adipocyte maintenance media (Zenbio catalog # AM-1); these were refreshed every 3–5 days for a further 18 days until assay at day 21 (Figure S1B). Lonza cells were treated with differentiation media and test chemicals for three days, then switched to adipocyte maintenance media (Lonza catalog # PT-3102A) for three days (Figure S1C). This cycle was repeated twice more (three days chemicals/differentiation media, then three days chemicals/adipocyte maintenance media) and then maintained in adipocyte maintenance media (media/chemical changes every 3–4 days) until assay at day 21.

### 2.3. Adipogenic Differentiation and Outcome Measurements

The plates were processed for measurements of triglyceride accumulation and DNA content, as described previously [48,49,50,51,52]. Briefly, the cells were rinsed with Dulbecco’s phosphate-buffered saline (DPBS) and then treated with 200 μL/well of a dye mixture: ~19 mL DPBS, 20 drops/mL NucBlue^®^ Live ReadyProbes^®^ Reagent (DNA content; Thermo cat # R37605) and 500 μL Nile Red solution (40 μg/mL solution; Sigma cat #72485-100MG). After the addition, the plates were protected from light and incubated for forty minutes, and then the fluorescence was measured using a Molecular Devices SpectraMax iD5 (San Jose, CA) at 485/572 nm excitation/emission for Nile Red and 360/460 for NucBlue. Triglyceride accumulation was reported as percent activity, corrected for intra-assay differentiated vehicle control responses and relative to the rosiglitazone-induced maximum. The DNA content was reported as percent activity relative to the differentiated vehicle control responses. Normalized triglyceride content was calculated as total triglycerides per well per unit DNA content (used as a proxy for triglycerides per cell). Four technical replicates (wells within each assay plate) and three biological replicates (separate cell passages/assays) were utilized for every test chemical and concentration.

### 2.4. Zebrafish Husbandry

Wildtype (AB) zebrafish (*Danio rerio*) were maintained according to protocols approved by the Wayne State University Institutional Animal Care and Use Committee, IACUC-20-06-2408. Breeding for embryo generation occurred following standard procedures [53]. Briefly, adult AB zebrafish were paired in breeding chambers overnight with gates pulled to initiate spawning the next morning at the time of lights on. Embryos were collected, cleaned, checked for viability, and stored overnight in embryo media (EM) with methylene blue. Embryos were fed beginning at 6 days post fertilization (dpf) with GEMMA Micro 75 (Skretting) twice daily until 15 dpf. At 15 dpf embryos were switched to GEMMA Micro 150 until 30 dpf.

### 2.5. Zebrafish Exposures

At 24 h post-fertilization (hpf), the embryos were staged, and viable embryos were separated into 50 mL glass crystallizing dishes in 10 mL EM for chemical exposures (*n* = 15 individual embryos/chemical test concentration). All exposures were performed in 10 mL EM using chemical stocks at 0.1% DMSO vehicle. Embryos were exposed from 24 hpf through 6 dpf with complete EM and tested for the chemical changes occurring daily. At 6 dpf, the media were replaced with fresh EM without test chemicals, and the embryos were aged to 30 dpf for morphometric measurements. Embryos were maintained in glass dishes in 15–30 mL of EM, until the time of sacrifice, with media changes occurring daily throughout the 30 days.

### 2.6. Zebrafish Metabolic Health

The alamar blue assay was used to measure zebrafish metabolic rate. The assay was performed at 6 dpf, according to previously published protocols [25,54]. Following chemical exposures, the zebrafish were transferred into fresh EM with no added chemicals. For metabolic testing, wells of *n* = 3 embryos were set up in 24-well black clear-bottom microtiter plates. For each test, chemical, control, and concentration, *n* = 3 wells were used per plate (*n* = 9 fish per exposure group, with two separate exposure experiments/biological replicates). Briefly, EM was removed from all wells and replaced with 1 mL of alamar blue working dye solution (99% embryo media, 1% alamarBlue Cell Viability reagent (Thermo cat# DAL1100)). Fluorescence was immediately measured using an iD5 Molecular Devices plate reader under 530/590 nm excitation/emission wavelengths, and then plates were incubated at 28 °C and protected from light. The fish were incubated overnight (approximately 16 h), and the fluorescence was then measured again; the change in fluorescence was calculated by the difference of the values at 16 h from those at the immediate read. The data are presented as the relative change in arbitrary fluorescence units normalized to the DMSO control animals.

### 2.7. Larvae Locomotion

At 6 dpf, the larval activity was assessed by using the swim distance in light and dark cycles, which was automatically quantified using Noldus Ethovision (version XT 16; Leesburg, VA, USA, [55]) during a 20 min period. Briefly, the larvae from the control and exposure groups were placed into a 24-well plate and were allowed to acclimate to a sound-insulated, temperature-controlled (26 °C), and light-controlled testing chamber, away from home tanks. All of the larvae were subjected to a 10 min period of light followed by a 10 min period of dark [56,57]. The movements of 24 individual larvae were simultaneously measured using an auto-detect feature of Ethovision, with all of the movement data then being binned into 60-s intervals. Manual observation of tracking success was conducted on at least two wells in each plate before any analysis. To reduce the potential for outlier observations, the data were smoothed using Ethovision before analysis. The raw data were exported into Microsoft Excel, and the average total distance moved (cm) per minute was analyzed in GraphPad Prism 9.0 (Boston, MA). The assay was replicated at least three times for each chemical and ethoxylate chain length, with each repetition performed on a different day with different larvae.

### 2.8. Morphology and Adipose Quantification

Following locomotor and alamar blue analyses, the fish were returned to glass dishes and maintained until 30 dpf. At 30 dpf, the fish were stained with a 0.5 μg/mL concentration of Nile Red for 30 min and protected from light. The fish were then euthanized with 150 mg/L tricaine (MS-222), mounted onto depression slides, and imaged using a Leica Thunder M205FA stereoscope. The fish were imaged under brightfield and a yellow fluorescent protein (YFP) filter at 2× magnification for full-body imaging and standard-length measurements. The fish were then imaged at 16× magnification for higher-resolution adipocyte fluorescence quantification. After imaging, the fish were blotted and weighed on a microbalance, then snap-frozen in liquid nitrogen. Body mass indices were calculated by converting weights to grams and dividing by the squared lengths in millimeters for each individual fish, as performed previously [25,58]. For standard length and adiposity quantifications, the files were imported into Fiji (version 2.1.0). The standard length of the fish was obtained from the images taken at 2× magnification by using the segmented line tool to trace the contour of the fish from the frontmost part of the mouth to the beginning of the caudal fin. Fiji was able to calculate the length of the line based on the internal scale of each individual image. For images taken at 16x magnification, an individual image thresholding was used to select a pixel intensity range that outlined Nile-Red-stained, fluorescent adipose tissue. Regions of interest were drawn around the adipose tissue if the pixel intensity range was unable to outline adipose tissue without also outlining confounding high-intensity pixels, such as eye shines or fluorescence reflected off of the swim bladder. The total adipose tissue (AT) area was calculated for every individual in each treatment group and concentration. Image thresholding based on pixel intensity was performed to delineate AT area. For ATs that did not touch, the threshold was manually set using the slider until the area approximated the lipid dye [39]. Following quantification, the 34 defined adipose depots [37,39] were scored as present or absent and then compared to controls to determine the potential dysregulation of specific adipose depots.

### 2.9. Statistical Analysis

The cell data are presented as means ± SEM from four technical replicates of three independent biological replicates. Zebrafish growth and metabolic data are presented as means ± SEM from 10–15 replicates (technical replicates from four independent spawning events/biological replicates). Non-normality was confirmed, and Kruskal–Wallis with Dunn’s multiple comparisons test was performed to determine significant differences across concentrations and relative to DMSO control fish (*p* < 0.05 considered significant). Statistical comparisons were made using GraphPad Prism 9.0. To address whether the outliers may influence the take-home findings from these experiments, a sensitivity analysis was also performed by removing the outliers with values greater than or equal to three standard deviations from the mean. These results are provided in the supplemental information and discussed in greater detail below.

## 3. Results

CetAEOs were assessed for adipogenic activity in vitro by utilizing one murine pre-adipocyte model and two hMSC models and for obesogenic activity in vivo by utilizing developmental exposures and growth measurements in zebrafish.

### 3.1. Adipogenic Activity of CetAEOs

**Figure 1:**
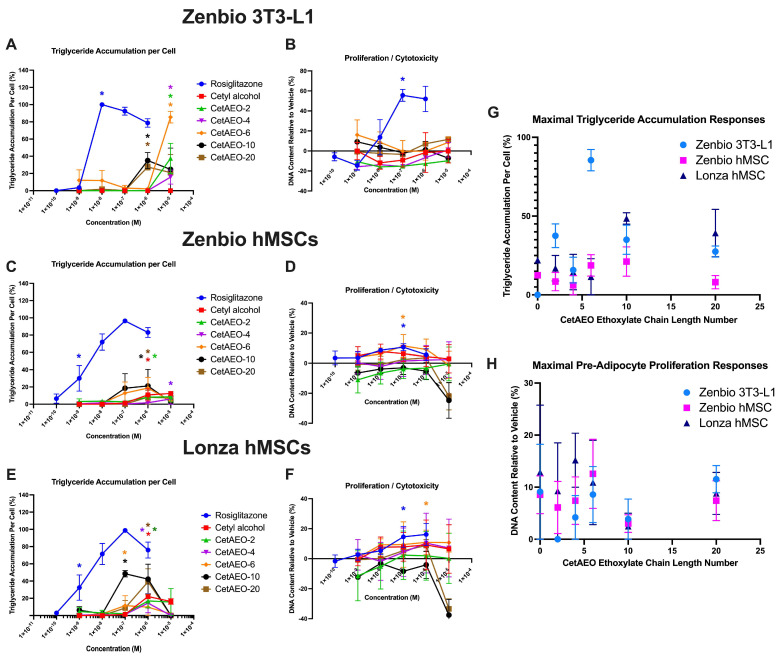
Cetyl alcohol and Polyethoxylates Promote Adipogenesis in Mouse Pre-adipocytes and Human Mesenchymal Stem Cell Models. Murine 3T3−L1 and both Zenbio and Lonza−sourced human bone marrow−derived mesenchymal stem cell models were differentiated as described in Methods and assessed for adipocyte differentiation (Nile Red staining of lipid accumulation) and cell proliferation (NucBlue^®^ Live ReadyProbes^®^ Reagent staining) after 10/21/21 (respectively) days of differentiation while exposed to controls chemicals as well as cetyl alcohol and its ethoxylates. Percent triglyceride accumulation per cell relative to maximal rosiglitazone response (normalized to DNA content) (**A**,**C**,**E**). Changes in DNA content (increase = cell proliferation, decrease = potential cytotoxicity) relative to vehicle control (**B**,**D**,**F**). Data presented as mean ± SEM from three independent experiments. * indicates lowest concentration with significant increase in triglyceride over vehicle control or cell proliferation/cytotoxicity relative to vehicle control, *p* < 0.05, as per Kruskal−Wallis in GraphPad Prism 9. Panel (**G**) provides a summary plot of maximal effects on triglyceride accumulation based on ethoxylate chain length across cell models, comparing results from panels (**A**,**C**,**E**). Panel (**H**) provides a summary plot of maximal effects on pre-adipocyte proliferation based on ethoxylate chain length across cell models, comparing results from panels (**B**,**D**,**F**). CetAEO = cetyl alcohol polyethoxylate (with varying average ethoxylate chain lengths).

Cetyl alcohol failed to induce any triglyceride accumulation in murine pre-adipocytes, though each of the varying chain length ethoxylates did (Figure 1A). Specifically, CetAEO-6 induced 85% triglyceride accumulation relative to the maximal rosiglitazone-induced response, with lower (35–40%) activity for CetAEO-2, 10, and 20. CetAEO-4 induced the lowest effects on triglyceride accumulation (~16%). Neither the CetAEOs nor the base alcohol were able to induce pre-adipocyte proliferation in 3T3-L1 cells (Figure 1B). In Zenbio-sourced hMSCs, cetyl alcohol induced 12% triglyceride accumulation relative to the maximal rosiglitazone-induced response at 10 mM (Figure 1A). In this model, CetAEO-6 and 10 induced the greatest degree of triglyceride accumulation (19% and 21%, respectively), with CetAEO-2 and 20 inducing approximately 11% (Figure 1C). CetAEO-6 also promoted significant proliferation (11%) relative to the differentiated solvent control responses (Figure 1D), equivalent to the rosiglitazone-induced response via this metric. In Lonza-sourced hMSCs, more potent and efficacious responses were observed relative to the Zenbio-sourced hMSCs. CetAEO-10 promoted the strongest effect (48% at 0.1 μM), with CetAEO-6 promoting 11% triglyceride accumulation at 0.1 μM as well (Figure 1E). At 1 mM, CetAEO-20, the base cetyl alcohol, CetAEO-2, and -4 promoted significant triglyceride accumulation (39%, 22%, 17%, and 14%, respectively). As with Zenbio-sourced hMSCs, CetAEO-6 was the only compound able to promote significant proliferation (Figure 1F). Maximal responses for each test chemical were further compared across each of the three adipogenesis models for triglyceride accumulation and proliferation. For triglyceride accumulation, responses were generally consistent (Figure 1G), with maximal responses occurring in medium-chain-length compounds (CetAEO-6 or -10) and with generally lower efficacies for the hMSCs relative to the 3T3-L1 model (with the exception of the longer chain length CetAEOs). This was generally consistent for proliferation responses (Figure 1H), though most of these responses were not significantly different from the baseline; as noted previously, only CetAEO-6 promoted significant effects in the hMSC models.

### 3.2. Lethality of NPEOs on Zebrafish

**Figure 2:**
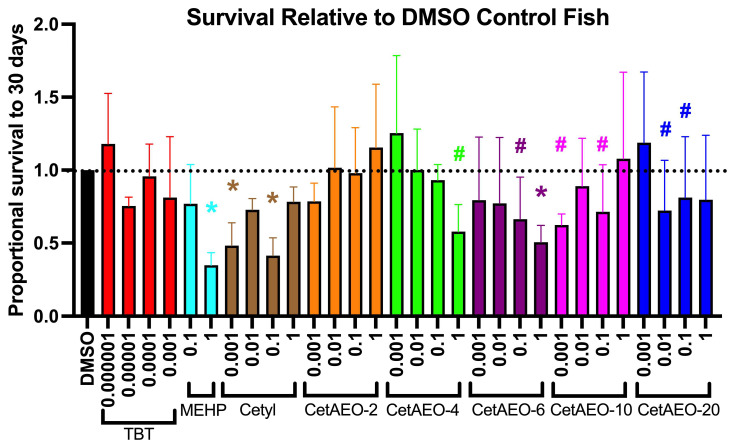
Lethality of Cetyl Alcohol and Polyethoxylates in Developmentally Exposed Zebrafish. Zebrafish were developmentally exposed to control chemicals, cetyl alcohol, or cetyl alcohol polyethoxylates from one through six days post fertilization. Following exposures, fish were aged to 30 days post fertilization. Lethality was measured daily to determine survivorship across test chemicals and treatments for each test chemical (A). N = 15 replicate fish in each biological replicate (spawning event) for each test chemical and concentration, and four spawns were performed (four biological replicates) and averaged for responses depicted here for approximately 60 fish evaluated per experimental group. Lethality is depicted as survival percent relative to DMSO vehicle control exposed fish at 30 days. All concentrations are provided in μM. * indicates significant change in survival compared to vehicle control fish, *p* < 0.05, as per Kruskal–Wallis test with Dunn’s multiple comparisons. # indicates 0.05 < *p* < 0.10. DMSO = dimethylsulfoxide, vehicle control; TBT = tributyltin chloride; MEHP = Mono(2−ethylhexyl) phthalate; CetAEO = cetyl alcohol polyethoxylates (with varying average ethoxylate chain lengths).

Zebrafish were exposed to test chemicals at a range of concentrations from approximately 24 h post-fertilization through to 6 days post-fertilization (dpf). Throughout and following the exposures, the zebrafish were checked daily for the lethality of the test chemicals (dead embryos removed to protect remaining live zebrafish) and to determine non-toxic concentrations for subsequent analyses. Both the vehicle (DMSO)-treated and control (embryo media, no exposure) fish had average survival rates of approximately 70% throughout the 30 days, with no appreciable mortality observed during the chemical exposure window and limited mortality in the weeks following. Chemical exposures were calculated relative to the DMSO-treated fish survival to account for this baseline mortality observed in our system. Each of the CetAEOs at 10 μM induced >75% mortality relative to the DMSO-treated fish and were thus removed from the study following preliminary dose-finding. The TBT positive control was significantly more toxic than CetAEOs, with complete lethality noted for concentrations of 0.01 μM and above (and thus excluded). At 1 μM, MEHP induced approximately 60% mortality (*p* < 0.05), CetAEO-4 promoted 40% mortality (*p* < 0.10), and CetAEO-6 promoted approximately 50% mortality (*p* < 0.05; Figure 2). Interestingly, cetyl alcohol had significant mortality in the 0.001 μM and 0.1 μM exposure groups (50% and 60% mortality, respectively; *p* < 0.05), despite having no significant effects at 0.01 μM and 1 μM. A similar pattern was observed for CetAEO-10, which had non-significant increases in mortality at 0.001 and 0.1 μM (40% and 30%, respectively; *p* < 0.10). CetAEO-6 also tended to exhibit mortality at the 0.1 μM dose (~30% mortality, *p* < 0.10). Lastly, CetAEO-20 tended to increase mortality at the 0.01 and 0.1 μM doses (28% and 20%, respectively; *p* < 0.10).

**Figure 3:**
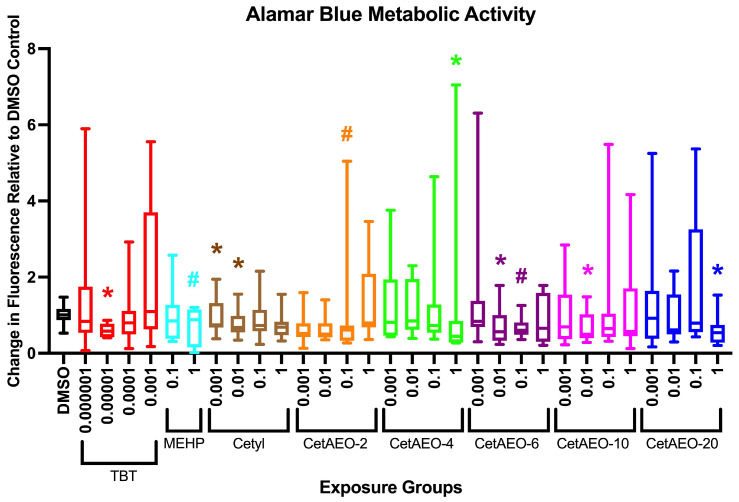
Metabolic Activity in Zebrafish Developmentally Exposed to Cetyl Alcohol and Polyethoxylates. Zebrafish were developmentally exposed to control chemicals, cetyl alcohol, or cetyl alcohol polyethoxylates. Immediately following exposure, at six days post fertilization, metabolic activity was measured using the alamar blue assay. Four groups of three replicate fish were transferred into wells of a 24−well black clear−bottom plate, media removed, and alamar blue solution added to wells. Plates were immediately read for fluorescence, then incubated in the dark overnight before measuring fluorescence again. The increase in fluorescence is correlated with increased metabolic activity in the fish. Chemical−exposure−treated fish responses were compared with DMSO (0.1%, vehicle control) −treated fish to determine significant differences. N = 9 replicate fish in each biological replicate (spawning event), and three spawns were performed for approximately 27 fish per exposure group. * indicates significant change in arbitrary fluorescence compared to vehicle control fish, *p* < 0.05, as per Kruskal–Wallis test with Dunn’s multiple comparisons. # represents 0.05 < *p* < 0.10. DMSO = dimethylsulfoxide, vehicle control; TBT = tributyltin chloride; MEHP = Mono(2−ethylhexyl) phthalate; CetAEO = cetyl alcohol polyethoxylates (with varying average ethoxylate chain lengths). Box and whisker plots depict the following metrics: whiskers represent 10–90th percentiles, box bounds represent the 25th to 75th percentiles, and the middle line represents the median. All concentrations are provided in μM.

*Energy Expenditure and Activity at 6 Days.* Energy expenditure was determined via the alamar blue assay as an approximate measure of zebrafish oxidative metabolism and cellular metabolic respiration (e.g., NADH_2_ production) [54,59]. The 0.00001 μM TBT control had significantly lower energy expenditure than the DMSO control animals (*p* < 0.05, Figure 3), and the 1 μM MEHP animals tended to be reduced as well (*p* < 0.10). The two lowest doses of cetyl alcohol (0.001 and 0.01 μM) also had lower energy expenditure, along with the 0.1 μM CetAEO-2 group, the 1 μM CetAEO-4 group, the 0.01 μM CetAEO-6 group, the 0.01 μM CetAEO-10 group, and the 1 μM CetAEO-20 group (*p* < 0.05 all). The 0.1 μM CetAEO-2 and 0.1 μM CetAEO-6 groups tended to be reduced as well (*p* < 0.10). While no groups were significantly elevated relative to the DMSO control animals, a number of groups had increased variance, with a subset of animals demonstrating much greater energy expenditure relative to the DMSO control animals. This was particularly apparent in the 0.000001 μM and 0.001 μM TBT groups and across many of the medium- and long-chain-length CetAEOs. Removing outliers resulted in similar results for energy expenditure testing. The groups retained significance, and increased significance was noted for the CetAEO-2 exposure groups and the 1 μM cetyl alcohol group (Figure S2).

**Figure 4:**
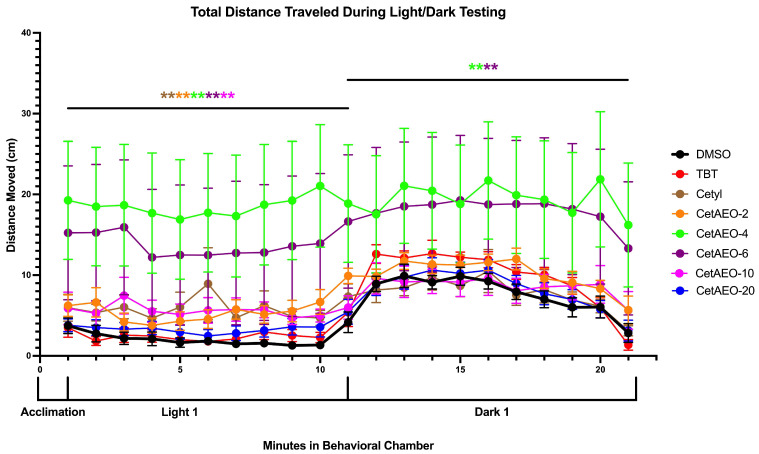
Total Distance Traveled During Light/Dark Neurodevelopmental Testing. Zebrafish were developmentally exposed to control chemicals, cetyl alcohol or cetyl alcohol polyethoxylates. Immediately following exposure, at six days post fertilization, activity was tracked using Noldus Ethovision software as described in Methods. Three replicate fish were transferred into wells of a 24-well clear-bottom plate, and the total activity was tracked using an acclimation period followed by one ten−minute light and then one ten−minute dark period. Experiment was completed at least three times (biological replicates). Total activity across the entire ten−minute period was compared between chemical exposures and DMSO (0.1%, vehicle control) treated fish to determine significant differences. ** indicates significant increase in activity compared to vehicle control fish, *p* < 0.01, as per Kruskal–Wallis test with Dunn’s multiple comparisons. DMSO = dimethylsulfoxide, vehicle control; TBT = tributyltin chloride; Cetyl = cetyl alcohol; CetAEO = cetyl alcohol polyethoxylates (with varying average ethoxylate chain lengths). Concentrations provided are the highest test concentrations for each treatment (1 μM for cetyl alcohol and ethoxylates and 0.001 μM for TBT). Full dose responses for each chemical are provided in Figure S2.

A separate set of fish was examined for the total activity at 6 dpf through light/dark photoperiod activity tracking. Following acclimation, activity in the DMSO-exposed fish remained low (~2 cm per minute) during the ten-minute light period and then sharply increased to approximately 10 cm/min during the subsequent ten-minute dark period (Figure 4). At 1 μM, cetyl alcohol, CetAEO-2, CetAEO-4, CetAEO-6, and CetAEO-10 displayed significantly greater activity throughout the light period relative to the DMSO control fish (*p* < 0.01), while TBT and CetAEO-20 showed no significant difference. During the dark period, CetAEO-4 and -6 displayed significantly greater activity relative to the control fish (*p* < 0.01; Figure 4). Interestingly, responses to CetAEO-4 and -6 were strikingly distinct from the other chemicals, maintaining a high degree of activity regardless of the light versus dark cycles. Testing was also performed across the full range of concentrations for each test chemical (Figure S3). While every other compound had effects for at least one concentration, no disruption of activity was observed for any concentration of TBT or CetAEO-20 tested (Figure S3A,G). Cetyl alcohol had significantly increased activity during the light period at 1 μM, 0.1 μM, and 0.001 μM (*p* < 0.01), and during the dark period only at 0.001 μM (*p* < 0.01; Figure S3B). CetAEO-2 had significantly increased activity at each concentration during the light period and only at 0.01 μM during the dark period (*p* < 0.01; Figure S3C). CetAEO-4 had significantly increased activity at each concentration during the light period and also for 0.001 μM, 0.1 μM, and 1 μM during the dark period (*p* < 0.01; Figure S3D). CetAEO-6 had significantly increased activity for 0.001 μM, 0.01 μM, and 1 μM during the light period and for 0.01 μM, 0.1 μM, and 1 μM during the dark period (*p* < 0.01; Figure S3E). Lastly, CetAEO-10 had significantly increased activity only at 1 μM during the light period, as noted above, with no effects observed during the dark period (*p* < 0.01; Figure S3F).

### 3.3. Growth Trajectory, Weights, and Adipose Deposition

**Figure 5:**
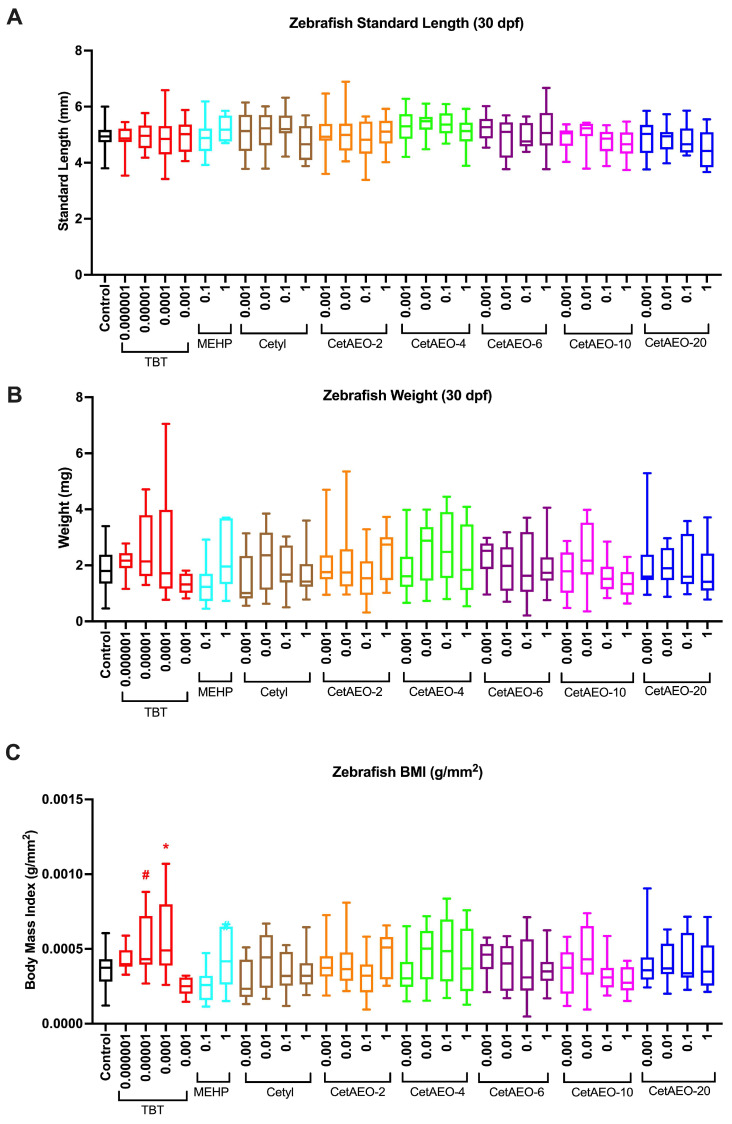
Growth Trajectories in Zebrafish Developmentally Exposed to Cetyl Alcohol and Polyethoxylates. Zebrafish were developmentally exposed to control chemicals, cetyl alcohol, or cetyl alcohol polyethoxylates; aged to 30 days post fertilization; then measured and stained with Nile Red according to Methods. Zebrafish were imaged, and the standard length of each fish was measured (**A**) using the integrated point−to−point measurement tool within the Leica software, which scales by magnification. Following imaging, zebrafish were blotted with Kimwipes and weighed on a microbalance to obtain total body weights (**B**) for each fish and then averaged across test chemicals and concentrations. Zebrafish body mass index or Fulton’s condition factor (**C**) was calculated by dividing the calculated standard length and weights and correcting measurement units to g/mm^2^. DMSO−exposed fish and embryo media fish were not significantly different and are thus included as one control reference group. N = 31 (controls), 14, 14, 15, 11, 17, 6, 13, 16, 15, 20, 14, 16, 17, 13, 17, 13, 11, 15, 11, 15, 11, 17, 11, 13, 15, 13, 15, 12, 12, and 11 across four spawning events (biological replicates) for exposure groups listed below, respectively. * indicates significant change over vehicle control fish, *p* < 0.05, as per Kruskal–Wallis test. Control = 0.1% dimethylsulfoxide and embryo media controls; TBT = tributyltin chloride; MEHP = mono(2−ethylhexyl) phthalate; CetAEO = cetyl alcohol polyethoxylates (with varying average ethoxylate chain lengths). # represents 0.05 < *p* < 0.10, as per statistics described above. Box and whisker plots depict the following metrics: whiskers represent 10−90th percentiles, box bounds represent the 25th to 75th percentiles, and the middle line represents the median. All concentrations are provided in μM.

Developmentally exposed zebrafish were aged to 30 dpf, stained with Nile Red, and then imaged, measured, and weighed. The zebrafish did not exhibit any significant changes in standard length across the treatment groups (Figure 5A), suggesting no gross impacts on body size due to the chemical treatments. There were also no significant differences in blotted weights at 30 dpf between the groups (Figure 5B). Despite this, there were appreciable increases in zebrafish body mass index (BMI; g/mm^2^) in relation to some control chemicals (Figure 5C). Specifically, TBT-exposed fish had increased BMI relative to the DMSO control fish (15% increase for 0.00001 μM TBT, *p* < 0.10; 31% increase for 0.0001 μM TBT, *p* < 0.05; 12% increase for 1 μM MEHP, *p* < 0.10).

**Figure 6:**
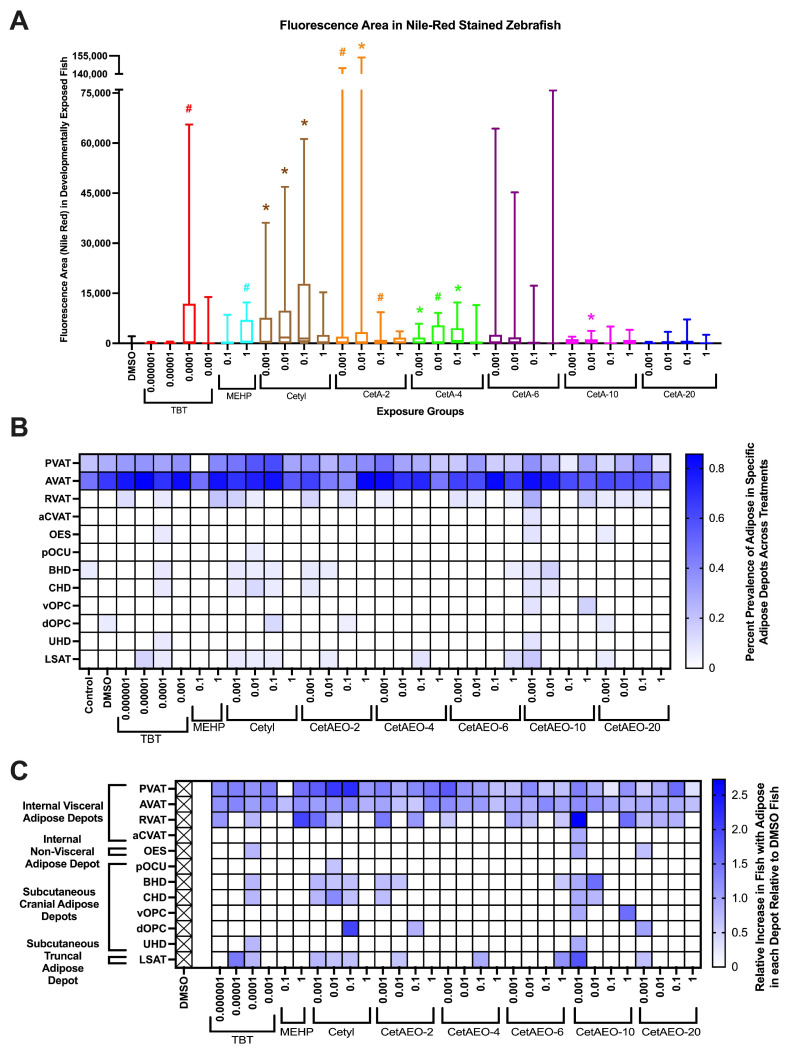
Adipose Deposition in Zebrafish Developmentally Exposed to Cetyl Alcohol and Polyethoxylates. Zebrafish were developmentally exposed to control chemicals, cetyl alcohol, or cetyl alcohol polyethoxylates (CetAEO−2−20), aged to 30 days post fertilization, and then measured and stained with Nile Red, as described in Methods. The area of total body fluorescence (μm^2^; (**A**)) was imaged at 16× magnification using a yellow fluorescent protein filter, and fluorescence was quantified for each fish, and then biological replicates were averaged. N = 16 DMSO, 14, 14, 15, 11, 17, 6, 13, 16, 15, 20, 14, 16, 17, 13, 17, 13, 11, 15, 11, 15, 11, 17, 11, 13, 15, 13, 15, 12, 12, and 11 across four spawning events (biological replicates) for exposure groups listed above, respectively. * indicates significant increase in total body fluorescence quantification over vehicle control fish, *p* < 0.05, as per Kruskal–Wallis test and # represents 0.05 < *p* < 0.10. Box and whisker plots depict the following metrics: whiskers represent 10–90th percentiles, box bounds represent the 25th to 75th percentiles, and the middle line represents the median. Developmental trajectory of adipose depots across the zebrafish (**B**,**C**). Percent of fish in each group with demonstrable fluorescing adipose within each exposure group (**B**) and relative proportions of fish exhibiting fluorescing adipocytes in each depot (**C**). Relative values in the DMSO fish were set as “normal”, and the heat map depicts increased or decreased proportions of fish in each group with visible adipocytes in each depot. Adipose depots labeled as per the developmental guides provided by Minchin and Rawls, 2017 (PMID: 28348140) and grouped based on anatomical classifications. A value of 2.5 represents a 2.5× increase in the proportion of fish in an exposure group with adipose in that particular adipose depot. PVAT = pancreatic visceral adipose tissue; AVAT = abdominal visceral adipose tissue; RVAT = renal visceral adipose tissue; aCVAT = anterior cardiac visceral adipose tissue; OES = oesophageal non-visceral adipose tissue; LSAT = lateral truncal adipose tissue; pOCU = posterior ocular adipose tissue; BHD = basihyal hyoid adipose tissue; CHD = ceratohyal hyoid adipose tissue; vOPC = ventral opercular adipose tissue; dOPC = dorsal opercular adipose tissue; and UHD = urihyal hyoid adipose tissue. Control = embryo media, no chemical or solvent exposures, DMSO = dimethylsulfoxide, vehicle control; TBT = tributyltin chloride; MEHP = mono(2−ethylhexyl) phthalate). All concentrations are provided in μM.

A quantitative assessment of the total body lipid accumulation across the test chemicals and concentrations in vivo was performed via fluorescent microscopy following Nile Red staining. The area of fluorescent adipose tissue was measured using ImageJ as pixels (Figure 6A) across the whole fish to determine the differences between the exposure groups. Manual scoring of the presence/absence of visible fluorescing adipocytes in each of the 34 characterized zebrafish adipose depots [37,39] was also performed to assess the location of any shifts in adipose deposition. The complete breakdown of adipose depot occurrence is provided in Figure 6B, with the relative occurrence (relative increase or decrease in treatments relative to DMSO control animals) provided in Figure 6C. The DMSO control fish had few apparent adipocytes by 30 days, primarily focused in the pancreatic and abdominal visceral adipose depots (~60% had visible adipocytes in these depots), though ~10% of the DMSO-exposed fish had visible adipocytes in some subcutaneous cranial adipose regions. Several significant differences were noted by fluorescent adipose tissue (FAT) area quantification. Specifically, 0.0001 μM TBT fish had increased total adipose relative to the control fish (*p* < 0.05; Figure 6A), and this appeared to be focused on the pancreatic, abdominal, and renal visceral (PVAT, AVAR, and RVAT, respectively) depots; the oesophageal non-visceral (OES) depot; the basihyal hyoid, ceratohyal hyoid, and urihyal (BHD, CHD, and UHD, respectively) depots; and the lateral truncal (LSAT) depot (Figure 6C). The 0.1 and 1 μM MEHP groups had increased adipose relative to the controls (*p* < 0.05), and this appeared to be mostly constrained to the PVAT, AVAT, and RVAT depots. Each concentration of cetyl alcohol (0.001–1 μM) had greater adipose relative to the controls, and this appeared to be spread across the PVAT, AVAT, RVAT, OES, BHD, CHD, and LSAT, as well as the posterior ocular (pOCU) and dorsal opercular (dOPC) depots. The 0.001 and 0.01 μM concentrations of CetAEO-2 had significantly increased FAT area relative to control fish, and this appeared to be concentrated in the PVAT, AVAT, RVAT, BHD, CHD, and dOPC depots. The 0.001–0.1 μM concentrations of CetAEO-4 had significantly increased FAT area, apparently constrained to the PVAT, AVAT, RVAT and LSAT depots. Lastly, the 0.01 and 1 μM CetAEO-10 fish had increased FAT area relative to the control fish, with the increased adipose seemingly focused in PVAT, AVAT, RVAT, BHD, CHD, and ventral opercular (vOPC) depots. Apparent shifts in adipose presence were observed across other groups (most notably for 0.001 μM CetAEO-10) and might reflect the re-distribution of adipose rather than increased deposition, as the FAT area was not significantly different for some of these other groups. Removing outliers resulted in similar results for adipose area quantification, with all exposure groups retaining the significant differences noted in the primary analysis of all exposed zebrafish (Figure S4).

## 4. Discussion

We previously published data using the 3T3-L1 murine pre-adipocyte model demonstrating a high magnitude of adipogenic activity for various alkylphenols, alcohols, and their polyethoxylates [24]. Further assessment of the NPEOs demonstrated consistent adipogenic effects in two separate human mesenchymal stem cell models [25] as well as pro-obesogenic effects following developmental exposures in zebrafish [25]. A purportedly lower toxicity replacement to NPEOs are AEOs, which have been suggested to degrade faster and into less toxic metabolites. Despite this, many of these AEOs demonstrate moderate to high aquatic toxicity. To determine whether the previous 3T3-L1 testing was robust, we herein characterized CetAEOs with a range of ethoxylation in three in vitro models and also in zebrafish. Herein, we report consistent pro-adipogenic effects in hMSCs and 3T3-L1 cells, which vary in terms of ethoxylate chain length, as well as metabolic disruption in vivo in the vertebrate zebrafish model (altered energy expenditure, total activity, and disrupted adipose development).

Several differences were noted between adipogenic activity testing across the models. Significant effects on triglyceride accumulation were noted for each CetAEO, with the maximal effects (~90% activity relative to the rosiglitazone-induced maximum) observed for CetAEO-6. No effects were observed for the base cetyl alcohol or for any of the compounds for the pre-adipocyte proliferation metric. Each of the CetAEOs induced significant triglyceride accumulation in both hMSC models, though to different maximal activities and at different concentrations than in 3T3-L1 cells. The effects were often higher potency (significant effects at lower concentrations) in hMSCs but with considerably lower magnitudes than in 3T3-L1 cells. Interestingly, consistent effects were observed in the hMSC models on the proliferative response, with CetAEO-6 promoting significant proliferation relative to the differentiated vehicle control cells. Consistency was also observed in patterning, with medium-chain-length AEOs inducing the highest magnitude effects across test chemicals, similar to what was observed for the NPEOs [25].

We report for the first time that cetyl alcohol and its ethoxylates induced metabolic health disruption in developmentally exposed zebrafish. Interestingly, we observed no significant changes in either standard length or total body weight. We did observe significant increases in zebrafish BMI (weight/length squared) for the positive control compounds TBT and MEHP, though still not for any of the ethoxylates examined. Our effects appeared to be more restricted to adipose deposition. Adipose area (as determined via Nile Red fluorescence) was significantly increased in TBT and MEHP control fish, as well as in multiple concentrations of the cetyl alcohol, CetAEO-2, CetAEO-4, and CetAEO-10 groups. There was increased variability in the CetAEO-6 group, with some fish showing greatly enhanced adipose, but these differences were not significantly different relative to controls. This is appreciably distinct from our previous study, where we saw more significant effects in our TBT-exposed animals [25]; though importantly, this was with a different source of fish, in a different facility, and on a different larval diet. While this could be that our assay for this set of chemicals was less sensitive than our previous testing, we retained significant effects in our control chemicals and thus suspect we would have observed effects for the CetAEOs if they were apparent. In addition, the increased activity we observed for many of our test compounds, even during the light cycles, supports the lack of increases observed in weights. In the case of the cetyl alcohols and ethoxylates, we seemed to see the most enhanced adipose deposition in subcutaneous cranial adipose depots. There was certainly enhanced visceral adiposity in the pancreatic, abdominal, and renal visceral depots, though this diversity was lower relative to the NPEOs we previously assessed. Instead, we saw a greater assortment of non-visceral depots occurring in the test chemical exposures in depots where no adipose was observed in our control animals. Interestingly, this increased adiposity, independent of changes in standard length or total body weight, suggests that there could be impacts on the recruitment of other cell lineages. This should be assessed further in future research.

We observed a particularly robust response to the light/dark testing performed in the exposed zebrafish at 6 dpf. Greatly elevated activity was observed in both the light and dark cycles across chemicals and concentrations, with the most striking effects observed for CetAEO-4 and CetAEO-6, where the larvae had no apparent response to light/dark changes and maintained a large increase in activity throughout the testing period. Increased activity was also observed for the base cetyl alcohol, CetAEO-2, and CetAO-10 during the light phase only, whereas CetAEO-20 did not impact activity in either phase at any concentration tested. Neurotoxicity has also been observed previously following exposure to a commercial AEO mixture, with the 0.8 and 3.2 μg/L concentrations reducing the distance traveled and total activity of the exposed zebrafish [60]. This was also demonstrated in another study examining commercial AEO mixtures used in lubricant emulsions [61]. A recent study examined a set of detergents (known to contain AEOs) and several linear AEOs and found that both the detergents and the AEOs were able to significantly reduce the mean swimming speed of zebrafish following exposures to 150 ppm detergents or 50 ppm AEOs [62]. In stark contrast, we observed significantly increased activity; this difference could be due to a number of factors. First, the timing of exposure in each of these three studies varied from ours, spanning embryonic to adult exposures, and varying stages of brain development. Second, the compositions and concentrations of the test chemicals varied between studies, with us testing more analytical mixtures as compared to more technical industrial mixtures employed in these other studies. We focused on a single alkyl chain length (cetyl, 16 carbons) and a range of ethoxylation (average of 0, 2, 4, 6, 10, and 20 ethoxylate chains), while these other studies assessed varying alkyl and ethoxylate chains. Third, large differences in the behavioral testing paradigm used between studies may have contributed to differences as well.

Interestingly, one-third to one-half of the animals in the medium-to-long-chain CetAEO groups demonstrated a drastic increase in activity under the light/dark neurodevelopmental testing. This same trend was observed in the alamar blue energy expenditure testing, with a subset of animals demonstrating a large increase in energy expenditure relative to the DMSO control animals. It could be that these increased energy expenditure and increased activity animals were a subset of animals demonstrating a differential response to the contaminants relative to the rest of the exposure groups. This should be examined further in future studies, as well as potential mechanisms for the variance in these responses.

Greater gross toxicity was observed for the CetAEOs relative to the NPEOs that we examined previously [25]. For the NPEOs, the 10 μM concentration was toxic for all but NPEO-20, with the 1 μM concentration exhibiting 20–30% mortality for the base nonylphenol as well as NPEO-2 and NPEO-4. The 10 μM concentration was overtly toxic for all CetAEOs and was thus excluded from our study. Significant toxicity of up to 50% relative to the DMSO control animals was observed for CetAEO-4 and -6 at 1 μM and for CetAEOs-6, -10, -20, and for the base cetyl alcohol at nM concentrations. A number of studies have previously reported a high degree of aquatic toxicity following CetAEO exposures in various organisms. Studies examining commercial AEO mixtures in *Xenopus* reported 72 h LC_50_ values of ~5 mg/L, various malformations (edema, loss of pigmentation, and microcephaly), and the collapse of the mitochondrial electrochemical gradient [63]. Various alcohol-based surfactants induced 96 h LC_50_ values of ~8 mg/L in bluegill sunfish [64] and ~3.0 mg/L 28-day toxicity in fathead minnows [65] (with more sensitive effects on growth than survival). A variety of AEOs induced no observed effect concentrations on survival and reproduction from 0.8 to 2.8 mg/L with varying carbon chain lengths of 10–14.5 (cetyl alcohol, examined here, has a backbone of 14 carbons) and average ethoxylate chains of ~6.5 in *Daphnia* [66]; 21-day LC50s were 1.2–5.9 mg/L for these surfactants [66]. To provide context, our observed effects generally occurred at 1 and 10 μM concentrations, which are approximately equivalent to concentrations of 0.33–3.3 mg/L for CetAEO-2 and up to 1.12–11.23 mg/L for CetAEO-20. Given the presumed lower toxicity of the AEOs and their metabolites, this increased toxicity should be evaluated further in future studies.

Studies have generally reported that the toxicity of AEOs increased with increasing alkyl chain length [67,68,69] across species, which we also found to be true for adipogenic responses previously [24]. They have also generally supported that average alkyl chain length had a greater impact on toxicity than average ethoxylate chain length [67,69], though this was species-specific. Interestingly, another study confirmed that toxicity increased with increasing alkyl chain length and showed a parabolic relationship with ethoxylate chain length (with a maximum at eight ethoxylate units) [68]; this is very similar to the results obtained in our study, where our maximum effects occurred around an ethoxylate chain length of six. Some reports have also suggested synergistic effects of AEOs on pesticide-induced toxicity against several pests and *Daphnia* [70,71], with synergism observed across multiple pesticide combinations, though antagonistic relationships observed for several [71], to an appreciably greater degree than the APEO alternatives. There has also been some toxicity testing in zebrafish previously. Specifically, AEOs were found to be quite toxic to zebrafish, with 48 h embryo and 96 h adult LC_50_ values of 5–6 mg/L [72]. Another study examining a commercial AEO mixture in zebrafish embryos reported an LC50 of ~15 μg/L, with detrimental effects on organ development at concentrations as low as ~3 μg/L (increased heart rate, reduced hemoglobin, increased liver size, increased total lipid retention) [60]. Another study of the AEO mixtures used in lubricants found that decreasing ethoxylation resulted in increased toxicity to zebrafish [61]. A recent study examined a set of detergents (known to contain AEOs) and several linear AEOs and found that both the detergents and the AEOs were able to significantly induce lethality in zebrafish larvae, with the AEO mixture demonstrating robust lethality even at the lowest concentration tested (50 ppm) [62]. These results raise concerns over the use of these surfactants to replace the APEOs.

Our preliminary assessment of cetyl alcohol and its ethoxylates suggests metabolic disruption potential that does not appreciably decrease with decreasing ethoxylate chain length. Similar to NPEOs, there was greater reported toxicity for the base cetyl alcohol, which the ethoxylates eventually degraded into. The CetAEOs are purported to have lower toxicity and degrade into less toxic metabolites, though our results suggest that this may not be entirely true. Interestingly, we saw perhaps the most striking effects on adipose deposition for the base cetyl alcohol and across the full range of concentrations, with significantly increased adipose even in the 0.01 μM exposure group. While we previously reported that internal visceral depots were more disrupted with the NPEO exposures relative to subcutaneous depots, this same trend was not necessarily observed for this set of contaminants. We saw greater diversity in the subcutaneous depots, though the visceral depots were certainly enhanced in the chemically exposed animals relative to the controls. Consistent with the NPEOs, we again observed the development of adipose depots long before their normal developmental timing. PVAT and AVAT are the first depots to develop in the zebrafish [37,38,39]. Other impacted depots (BHD, CHD, RVAT) are also earlier developing depots, though here we observed their development often before their standard developmental length (as a measure of developmental timepoint). Other depots, such as UHD and dOPC, developed in some exposed fish long before they should normally have developed. These results describe a need for future assessments that further elucidate depot-specific effects following exposure to adipogenic chemicals.

Interestingly, we observed different impacts on endpoints comparing these AEOs to APEOs [25]. In vitro, we observed the same pattern of adipogenic activity as we had observed for the NPEOs, with maximal activity observed in the medium-chain-length ethoxylates. We have also reported extremely high adipogenic activity and toxicity for a novel set of fluorotelomer ethoxylates found in commercial products [73], which should also be explored further in vivo. The medium-chain-length AEOs had the greatest impacts in the neurodevelopmental testing (based on activity during the light/dark testing) and in the alamar blue energy expenditure testing. However, the greatest impacts on adipose deposition were observed in the base cetyl alcohols and the lower-chain-length ethoxylates (CetAEO-2, 4). The mechanisms underlying these effects and underlying the differences between the NPEOs and the CetAEOs should be assessed further in future studies [24].

## 5. Conclusions

In summation, we report obesogenic effects in our zebrafish model and in multiple in vitro models for these cetyl alcohol polyethoxylates. These compounds are NPEO alternatives, suggesting that they may be a “regrettable substitution”. There is high conservation across vertebrates for adipose morphology, energy storage and lipid depot development, and associated/underlying gene signaling [37,38,39,74]; as such, these results, coupled with testing in human cell models, suggest a potential underexplored human health risk that necessitates further investigation into whether these chemicals may exacerbate obesity or contribute to the ongoing metabolic disorder pandemic [44,45,46]. Importantly, none of the ethoxylates included here have commercially available pure standards; instead, we have utilized commercial mixtures with average ethoxylate chain lengths. This limits the analytical characterization of these chemicals in dosing media and tissues and limits the environmental characterization of relevant concentrations, particularly for the longer-chain-length ethoxymers. There is a growing use of these and similar alcohol ethoxylates as replacement products following recommendations by the US Environmental Protection Agency [4]. Information regarding the toxicity of these compounds is much more limited and requires further evaluation in future studies.

## Figures and Tables

**Table 1:** Alcohol Ethoxylates and Control Chemicals.

Chemical	Acronym	CAS #	Manufacturer	Catalog #	Avg MW	Molecular Formula	Conc. Tested
**Alcohols/ethoxylates**							
cetyl alcohol	CetAEO (0)	36653-82-4	Chem Service	N-11416-1G	242.5	C_16_H_33_OH	1 nM–10 μM (vitro) 1 nM–1 μM (vivo)
cetyl alcohol ethoxylate (1–2)	CetAEO (2)	N/A	Sigma	388831-100G	330	C_16_H_33_(OCH_2_CH_2_)_2_OH	1 nM–10 μM (vitro) 1 nM–1 μM (vivo)
cetyl alcohol ethoxylate (4)	CetAEO (4)	N/A	Parchem	Ceteth-4	419	C_16_H_33_(OCH_2_CH_2_)_4_OH	1 nM–10 μM (vitro) 1 nM–1 μM (vivo)
cetyl alcohol ethoxylate (6)	CetAEO (6)	N/A	Barnet	BC-5.5	507	C_16_H_33_(OCH_2_CH_2_)_6_OH	1 nM–10 μM (vitro) 1 nM–1 μM (vivo)
cetyl alcohol ethoxylate (10)	CetAEO (10)	N/A	Barnet	BC-10	683	C_16_H_33_(OCH_2_CH_2_)_10_OH	1 nM–10 μM (vitro) 1 nM–1 μM (vivo)
cetyl alcohol ethoxylate (20)	CetAEO (20)	N/A	Chem Service	NG-S317-1G	1123	C_16_H_33_(OCH_2_CH_2_)_20_OH	1 nM–10 μM (vitro) 1 nM–1 μM (vivo)
**Control chemicals**							
Tributyltin chloride	TBT	1461-22-9	Sigma	442869	325.5	[CH_3_(CH_2_)_3_]_3_SnCl	1 pM–1 nM (vivo)
Mono(2-ethylhexyl) Phthalate	MEHP	4376-20-9	Santa Cruz Biotechnology	sc-396467	278.3	C_16_H_22_O_4_	0.1–1 μM (vivo)
Dimethylsulfoxide	DMSO	67-68-5	Sigma	34869-100mL	78.1	(CH_3_)_2_SO	0.1% as vehicle

Chemical identification, ordering information, and basic physicochemical properties for each of the alcohols, ethoxylates and control chemicals examined in this study. Molecular formulae contain base carbon number as well as average ethoxylate chain number.

## Data Availability

The data presented in this study are available in both this article and in supplementary material.

## Supplementary Materials

The following supporting information can be downloaded at: https://www.mdpi.com/article/10.3390/metabo13030359/s1, Figure S1: Adipogenic Assessment Models; Figure S2: Alamar Blue Metabolic Activity Testing; Figure S3: Total Distance Traveled During Light/Dark Neurodevelopmental Testing; Figure S4: Adipose Area Quantification in Zebrafish Developmentally Exposed to Cetyl Alcohol and Polyethoxylates.
