# Supplementary material for: Cetyl Alcohol Polyethoxylates Disrupt Metabolic Health in Developmentally Exposed Zebrafish
Source: Metabolites. 2023 Feb 28;13(3):359. doi: 10.3390/metabo13030359 (PMC10057089; doi:10.3390/metabo13030359)
Supplement: Supplementary file 1 [file metabolites-13-00359-s001.zip › metabolites-2225926-supplementary.pdf]

metabolites

MDPI

Supplemental Information

Contents

Figure S1, Adipogenic Assessment Models Page S1

Figure S2, Alamar Blue Metabolic Activity Testing Page S2

Figure S3, Total Distance Traveled During Light/Dark Neurodevelopmental Testing Page S3

Figure S4, Adipose Area Quantification in Zebrafish Developmentally Exposed to Cetyl Alcohol and Polyethoxylates Page S4

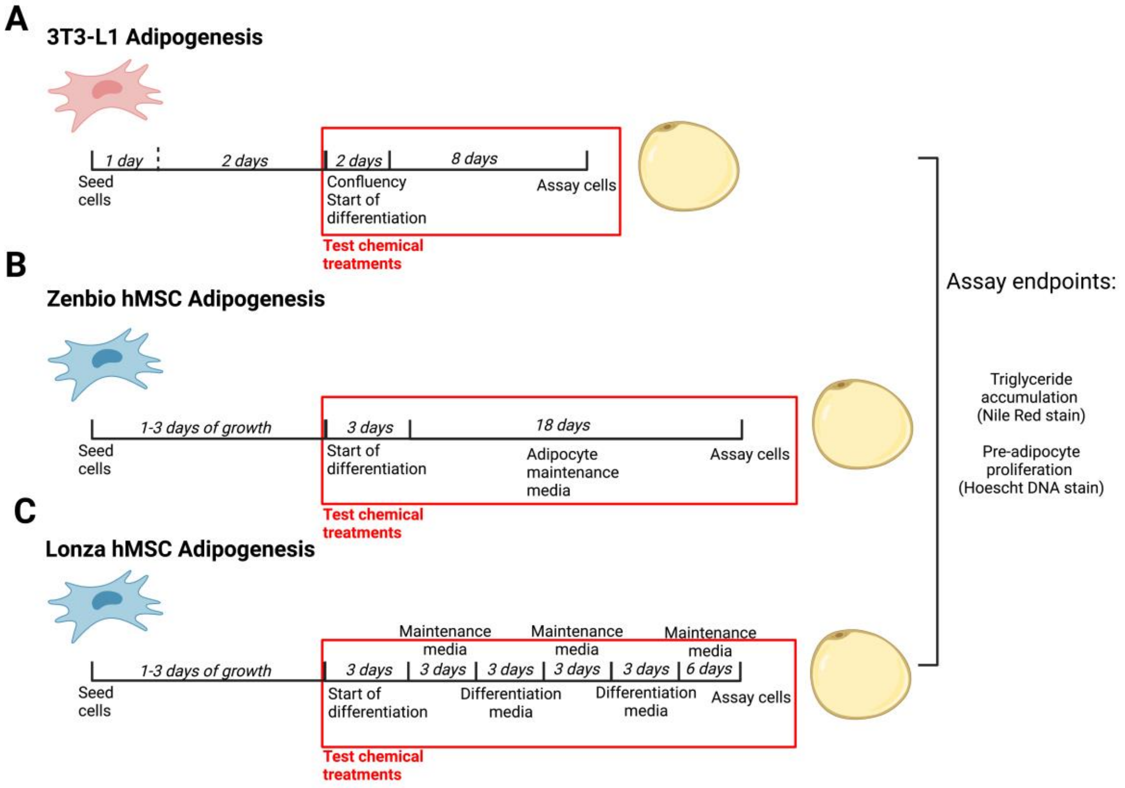

Figure S1. Adipogenic Assessment Models. Schematics of the adipogenic differentiation protocol followed for the murine 3T3-L1 cells (A) and the human bone marrow derived mesenchymal stem cells from Zenbio (B) and from Lonza (C). Protocols are performed as we have previously published and as described as optimal procedures by the manufacturers (particularly for mesenchymal stem cell models). Test chemicals and concentrations are provided in Table 1. Nile Red stain used for triglyceride accumulation measurements; NucBlue Live Cell Stain used for Hoescht DNA quantification measurements.

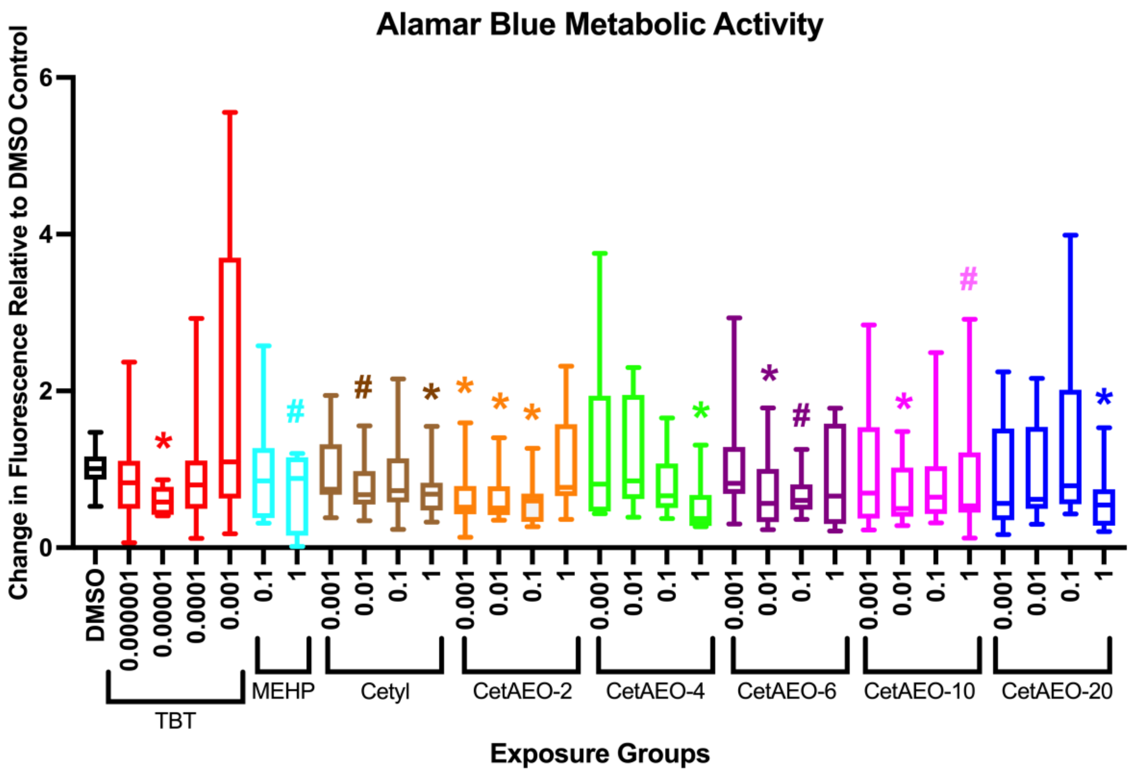

Figure S2. Alamar Blue Metabolic Activity. Zebrafish were developmentally exposed to control chemicals, cetyl alcohol, or cetyl alcohol polyethoxylates. Immediately following exposure, at six days post fertilization, metabolic activity was measured using the alamar blue assay. Four groups of three replicate fish were transferred into wells of a 24-well black clear-bottom plate, media removed, and alamar blue solution added to wells. Plates were immediately read for fluorescence, then incubated in the dark overnight, before measuring fluorescence again. The increase in fluorescence is correlated with increased metabolic activity in the fish. Chemical exposure treated fish responses were compared with DMSO (0.1%, vehicle control) treated fish to determine significant differences. N= 9 replicate fish in each biological replicate (spawning event), and three spawns were performed for approximately 27 fish per exposure group. * indicates significant change in arbitrary fluorescence compared to vehicle control fish, $p<0.05$, as per Kruskal-Wallis test with Dunn's multiple comparisons. # represents $0.05 < p < 0.10$. DMSO = dimethylsulfoxide, vehicle control; TBT = tributyltin chloride; CetAEO = cetyl alcohol polyethoxylates (with varying average ethoxylate chain lengths). Box and whisker plots depict the following metrics: whiskers represent 10-90th percentiles, box bounds represent the 25th to 75th percentiles, and the middle line represents the median. Outliers (values greater than or equal to three standard deviations from the mean) were removed as a sensitivity analysis. All concentrations are provided in µM.

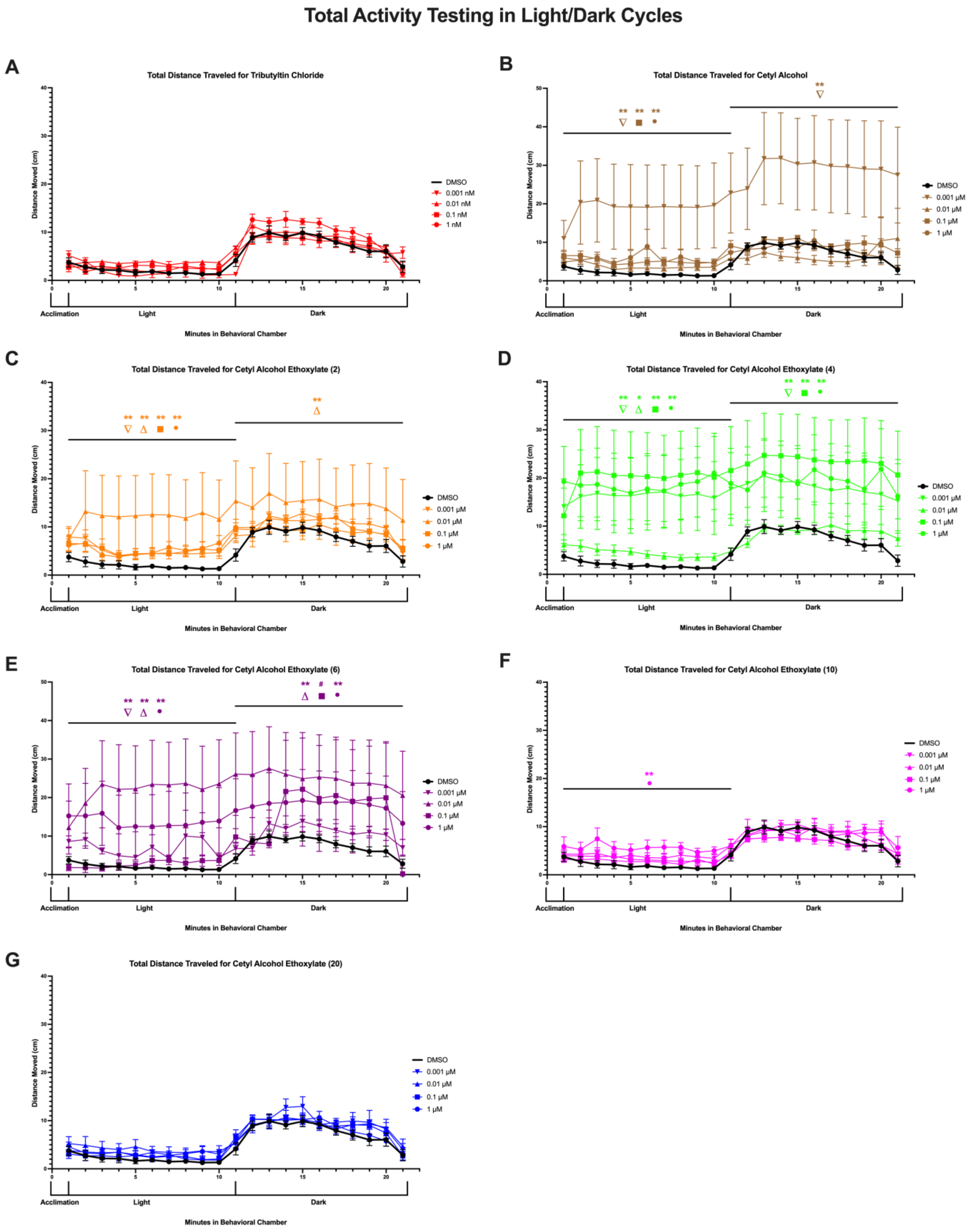

Figure S3. Total Distance Traveled During Light/Dark Neurodevelopmental Testing. Zebrafish were developmentally exposed to control chemicals, cetyl alcohol, or cetyl alcohol polyethoxylates. Immediately following exposure, at six days post fertilization, activity was tracked using Noldus Ethovision software as described in Methods. Three replicate fish were transferred into wells of a 24-well clear-bottom plate and total activity tracked using an acclimation period followed by one

ten-minute light and then one ten-minute dark period. Experiment was completed at least three times (biological replicates). Total activity across the entire ten-minute period was compared between chemical exposures and DMSO (0.1%, vehicle control) treated fish to determine significant differences for (A) each concentration of TBT (tributyltin chloride); for (B) each concentration of cetyl alcohol; for (C) each concentration of CetAEO-2 (cetyl alcohol ethoxylate with average ethoxylation of 2); (D) for CetAEO-4; (E) for CetAEO-6; (F) for CetAEO-10; and (G) for CetAEO-20. ** indicates significant increase in activity compared to vehicle control fish, $p<0.01$, as per Kruskal-Wallis test with Dunn's multiple comparisons. * indicates $p<0.05$ and # indicates $0.05<p<0.10$. Shapes correspond to the concentrations of test chemicals; circle depicts the high dose, square the second highest, triangle the third highest, and upside-down triangle the lowest.

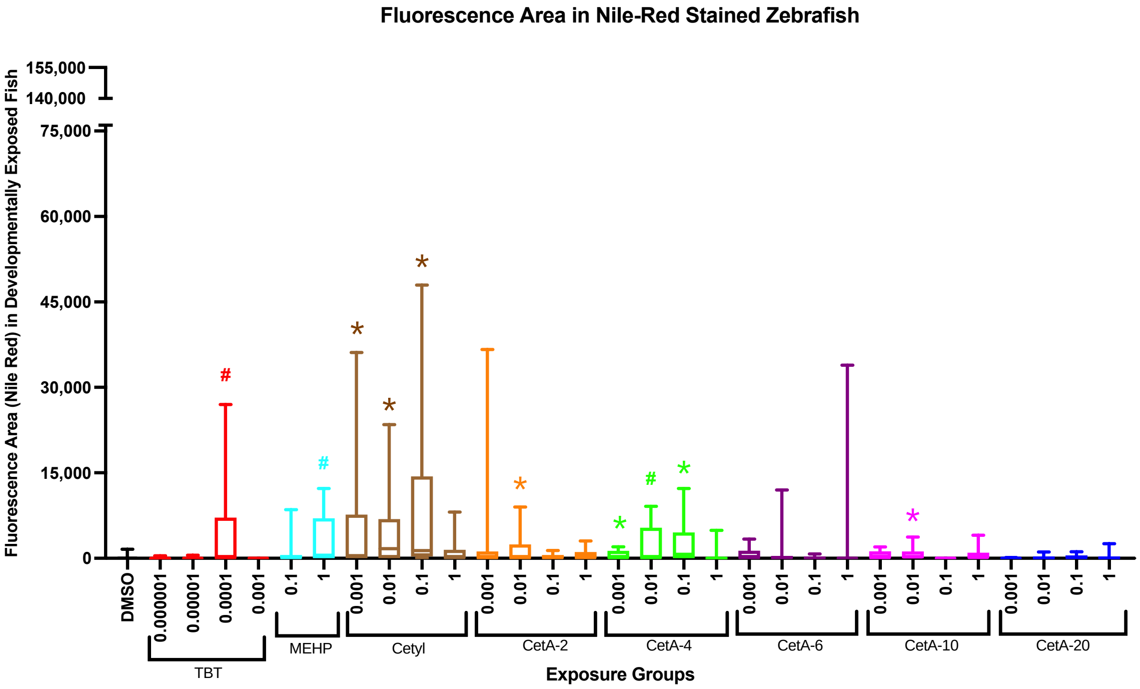

Figure S4. Adipose Area Quantification in Zebrafish Developmentally Exposed to Cetyl Alcohol and Polyethoxylates. Zebrafish were developmentally exposed to control chemicals, cetyl alcohol, or cetyl alcohol polyethoxylates (CetAEO2-20); aged to 30 days post fertilization; then measured and stained with Nile Red as described in Methods. Area of total body fluorescence (μm^2; A) was imaged at 16x magnification using a yellow fluorescent protein filter and fluorescence was quantified for each fish and then biological replicates were averaged. N= 16 DMSO, 14, 14, 15, 11, 17, 6, 13, 16, 15, 20, 14, 16, 17, 13, 17, 13, 11, 15, 11, 15, 11, 17, 11, 13, 15, 13, 15, 12, 12, and 11 across four spawning events (biological replicates) for exposure groups listed above, respectively. * indicates significant increase in total body fluorescence quantification over vehicle control fish, $p<0.05$, as per Kruskal Wallis test and # represents $0.05 < p < 0.10$. Box and whisker plots depict the following metrics: whiskers represent 10-90th percentiles, box bounds represent the 25th to 75th percentiles, and the middle line represents the median. DMSO = dimethylsulfoxide, vehicle control; TBT = tributyltin chloride; MEHP = mono(2-ethylhexyl) phthalate). Outliers (values greater than or equal to three standard deviations from the mean) were removed as a sensitivity analysis. All concentrations are provided in μM.
